# Role of serum neuron-specific enolase levels in the early diagnosis and prognosis of sepsis-associated encephalopathy: a systematic review and meta-analysis

**DOI:** 10.3389/fneur.2024.1353063

**Published:** 2024-02-29

**Authors:** MengQin Pei, YuShen Yang, ChunYan Zhang, QiaoMei Huang, YuMing Fang, LiMing Xu, Shu Lin, HeFan He

**Affiliations:** ^1^Department of Anesthesiology, The Second Affiliated Hospital of Fujian Medical University, Quanzhou, China; ^2^Centre of Neurological and Metabolic Research, The Second Affiliated Hospital of Fujian Medical University, Quanzhou, China; ^3^Diabetes and Metabolism Division, Garvan Institute of Medical Research, Sydney, NSW, Australia

**Keywords:** sepsis-associated encephalopathy, neuron-specific enolase, meta-analysis, biomarker, serum

## Abstract

**Background:**

Sepsis-associated encephalopathy (SAE) is one of the most ubiquitous complications of sepsis and is characterized by cognitive impairment, poor prognosis, and a lack of uniform clinical diagnostic criteria. Therefore, this study investigated the early diagnostic and prognostic value of serum neuron-specific enolase (NSE) in SAE.

**Methods:**

This systematic review and meta-analysis systematically searched for clinical trials with serum NSE information in patients with sepsis in the PubMed, Web of Science, Embase, and Cochrane databases from their inception to April 10, 2023. Included studies were assessed for quality and risk of bias using The Quality Assessment of Diagnostic Accuracy-2 tool. The meta-analysis of the included studies was performed using Stata 17.0 and Review Manager version 5.4.

**Findings:**

Eleven studies were included in this meta-analysis involving 1259 serum samples from 947 patients with sepsis. Our results showed that the serum NSE levels of patients with SAE were higher than those of the non-encephalopathy sepsis group (mean deviation, MD,12.39[95% CI 8.27–16.50, Z = 5.9, p < 0.00001]), and the serum NSE levels of patients with sepsis who died were higher than those of survivors (MD,4.17[95% CI 2.66–5.68, Z = 5.41, p < 0.00001]).

**Conclusion:**

Elevated serum NSE levels in patients with sepsis are associated with the early diagnosis of SAE and mortality; therefore, serum NSE probably is a valid biomarker for the early diagnosis and prognosis of patients with SAE.

**Systematic review registration:**

This study was registered in PROSPERO, CRD42023433111.

## 1 Introduction

Sepsis is a complex disease that develops as the host response to infection becomes dysregulated and is associated with acute organ dysfunction and a high risk of death (1). Sepsis is responsible for one-third of hospital deaths, due to its high incidence in intensive care units (ICUs) (2). Sepsis is associated with poor survival and prognosis due to the occurrence of multiple organ dysfunction, and the brain is often considered the first organ to be affected by an impaired inflammatory response (3). Therefore, the early recognition and diagnosis of sepsis-associated encephalopathy (SAE), a brain injury that is triggered by a host infection without an obvious central nervous system infection, is crucial. Up to 70% of patients with sepsis have various degrees of brain dysfunction, which is clinically characterized by cognitive impairment, drowsiness, confusion, delirium, and coma (4). Furthermore, SAE is strongly associated with increased ICU mortality, longer hospital stays, and greater utilization of ICU resources (5). Therefore, the early diagnosis and timely intervention of SAE in patients with sepsis are crucial for attaining better prognoses.

There are currently no uniform clinical diagnostic criteria for SAE. The final clinical diagnosis remains a diagnosis of exclusion. The diagnostic process for SAE involves first assessing the patient’s state of consciousness and degree of coma using non-specific score screening scales, namely, the Glasgow Coma Scale and the Confusion Assessment Method for ICU. A neurological examination is then performed to assess the central nervous system damage. Finally, a diagnosis of SAE is attained by excluding primary, metabolic, and neurological diseases caused by drugs or other causes (6). Therefore, the existing diagnostic methods for SAE are inefficient and have poor specificity. Recently, researchers have proposed electroencephalogram (EEG)-based diagnostic methods for SAE, due to their high sensitivity for early SAE identification and simplicity (7, 8). However, individual differences limit the role of EEG in the diagnosis of SAE; therefore, a rapid and reliable diagnostic method is needed.

Biomarkers, as relatively stable and easily measurable objective indicators of disease status, are a promising research direction (9). Currently, several biomarkers, such as neurofilament, S-100β, and C-type natriuretic peptides, are used to evaluate SAE (10, 11). Neuron-specific enolase (NSE), which is found in the cytoplasm of neurons, is of particular interest as a typical marker of brain injury. It is a cell-specific isoenzyme of the glycolytic enzyme enolase (12). Under normal physiological conditions, serum NSE levels are low, and the release of NSE into the cytoplasm after neuronal injury leads to a significant concurrent increase in serum NSE levels (13). Additionally, NSE concentration positively correlates with the degree of brain injury, and NSE also plays a vital role in small-cell lung cancer, cardiac arrest, and traumatic brain injury (14–16). The purpose of this systematic review and meta-analysis was to evaluate the role of serum NSE levels in the early diagnosis and prognosis of SAE.

## 2 Materials and methods

### 2.1 Search study

All relevant studies published before April 10, 2023 were included from the following databases: PubMed, Web of Science, Embase, and Cochrane. Titles, abstracts, or medical subject headings were searched for: (“Phosphopyruvate Hydratase” or “neuron-specific enolase” or “2-Phospho-D-Glycerate Hydrolase” or “gamma-Enolase” or “Nervous System-Specific Enolase” or “Non-Neuronal Enolase”) AND (“Sepsis” or “Severe Sepsis”); no restrictions on language were applied. Our study conforms with the Preferred Reporting Items for Systematic Reviews and Meta-Analyses (PRISMA) 2020 guidelines (17).

### 2.2 Selection criteria

**Figure 1:**
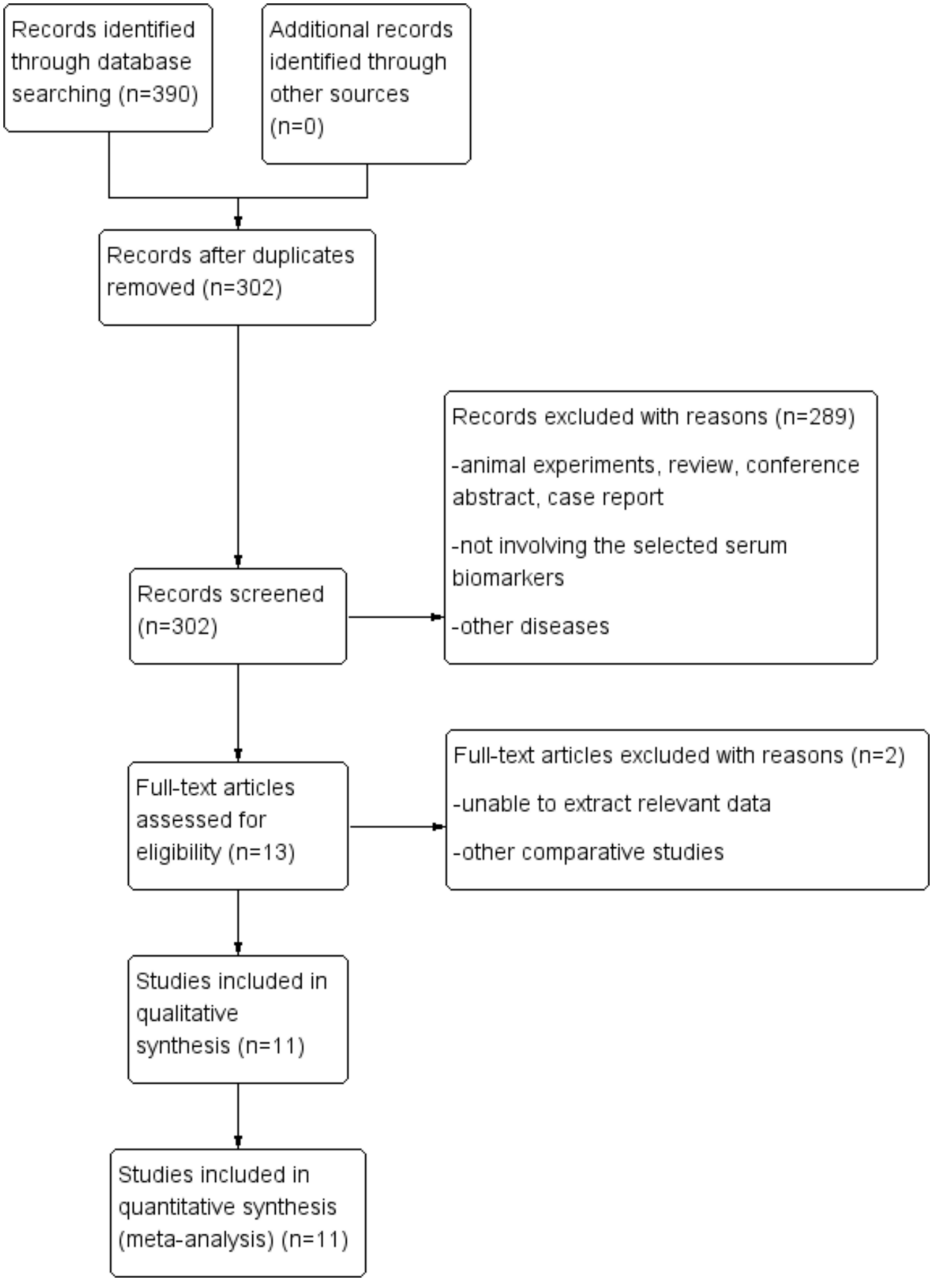
Study selection process.

Two reviewers independently used ENDNOTE X9 to screen the titles and abstracts of all retrieved documents and evaluate whether the full texts met the inclusion requirements. The final documents were decided upon through discussion, consultation, and, if necessary, third-party voting. The documents included in our analysis were determined prior to data extraction. The citation screening and selection process, which was based on the PRISMA standard flowchart, is shown in Figure 1. All selected studies met the following inclusion criteria: (1) all participants met the diagnostic criteria for sepsis; (2) only two groups were included in the study – one was the SAE and non-encephalopathy sepsis (NE) group, and the other was the survival and death group; (3) all experiments included in the analysis tested serum NSE indices; and (4) the studies were observational – either prospective or retrospective. The following exclusion criteria were used: (1) duplicate literature from different databases; (2) experimental observations and groupings unrelated to disease that did not meet the inclusion criteria; (3) animal experiments, reviews, conference abstracts, and case reports; and (4) serum NSE levels not recorded.

### 2.3 Data extraction

Two reviewers separately extracted the following information from the included studies: first author and year, clinical trial design type, SAE/death sample size, NE/survival sample size, age, sample collection time, and SAE/death NSE cutoff values. When there were inconsistencies in the extracted data, we decided on the more relevant datapoint through discussion. If necessary, a third reviewer was asked to finalize decisions. Serum NSE levels were standardized to ng/mL. If necessary, we attempted to contact the authors of the relevant studies directly to acquire relevant details about the data. When there were multiple serum NSE sample collection times in the same study, we used the corresponding order or time to differentiate, such as in El Shimy (1) and El Shimy (2) and Li (12 h) and Li (24 h).

### 2.4 Quality and risk assessment

We assessed study quality and risk of bias using the Quality Assessment of Diagnostic Accuracy-2 (QUADAS-2) tool (18). The QUADAS-2 tool comprises the following key domains: patient selection, index testing, reference standards, flow, and time risks. Two reviewers independently used the tool for assessments, and all authors discussed the findings and resolved any disagreements. We analyzed both the risk of bias and applicability, each of which had a corresponding question in the QUADAS-2 tool. Based on the answers to these questions, the risk of bias was identified as low, high, or uncertain for each aspect.

### 2.5 Statistical analysis

The mean deviation (MD) was used as a valid indicator of the continuous variables. When mean and standard deviation were not provided in the relevant literature, medians and interquartile ranges were used to estimate the means and standard deviations, using the methods of Luo et al. (19) and Wan et al. (20). *I*^2^ statistics and the *Q* test were used to evaluate the effect of heterogeneity between studies on the results of the meta-analysis (21). If *p* < 0.1 or *I*^2^ > 50%, there was significant heterogeneity, and we selected a random effects model for the meta-analysis. Begg’s and Egger’s tests were used to evaluate publication bias. Sensitivity analysis was performed to determine the stability of the meta-analysis results. For publication bias analysis, *p* > 0.05 indicated no significant publication bias; otherwise, the publication biases were considered significant. For the other analyses, *p* < 0.05 was considered to indicate statistical significance.

## 3 Results

### 3.1 Search results

Altogether, 390 studies were retrieved (PubMed, 78; Web of Science, 114; EMBASE, 192; and Cochrane Library, 6). Of these, 88 were excluded due to duplication, two were excluded owing to a lack of specific values of serum NSE or difficulties in data extraction, and 289 met the exclusion criteria after title and abstract screening. Ultimately, 11 studies met the inclusion criteria, overall encompassing 947 patients and 1,259 serum NSE samples. Further details of the included studies are provided in Table 1 (22–32).

**Table 1:** Characteristics of the included studies.

Study and Year	Design	SAE/Death sample size (Males/Females)	NE/Survival sample size (Males/Females)	Age	Sample Collection time	SAE/Death NSE cutoff (ng/mL)	Study location
de Araújo 2022	Prospective observational study	7 (−)	20 (−)	Children	1 d–7 d	4.02193 ± 0.652890	Rio de Janeiro, Brazil
El Shimy 2018	Prospective observational cohort study	34 (−)	62 (−)	Neonates	After birth; follow-up	cNSE:150 (92.38–190.5) Follow-up NSE:93.15 (65.65–102.95)	Cairo, Egypt
Erikson 2019	Prospective observational study	10 (4/6)	12 (10/2)	SAE:62.4 (49–70.5) NE:61.8 (60.1–78.5)	When CAM-ICU assessed	23.0 (13.2–28.0)	Oulu, Finland
Feng 2017	Retrospective study	36 (21/15)	23 (14/9)	SAE:52 ± 14 NE:57 ± 15	1 d, 3 d	1 d:19.28 (13.00, 30.52) 3 d:16.03 (9.40, 21.29)	Changsha, China
Guo 2021	NA	30 (17/13)	90 (42/48)	SAE:57.61 ± 4.16 NE:56.91 ± 4.85	NA	10.16 ± 2.11	Chenzhou, China
Li 2022	Retrospective study	21 (13/8)	20 (12/8)	SAE:37 ± 5 NE:38 ± 4	12, 24, 48 h	12 h:18.4 ± 2.2 24 h:26.3 ± 1.8 48 h:21.8 ± 2.0	Zhengzhou, China
Yan 2019	Retrospective study	58 (44/14)	94 (60/34)	SAE:55.8 ± 16.4 NE:55.0 ± 18.3	within 24 h	24.4 (15.7, 37.5)	Changsha, China
Yao 2014	Prospective observational study	48 (33/15)	64 (40/24)	SAE:56 ± 16 NE:52 ± 17	within 24 h	24.87 (31.73–12.73)	Changsha, China
Zhang 2016	Prospective observational study	29 (20/9)	28 (13/15)	SAE:55.55 ± 12.72 NE:56.21 ± 12.85	within 24 h	43.92 ± 14.66	Changsha, China
Zhang 2022	Prospective observational study	Death: 18(13/5)	Survival: 57(41/16)	Death:75.72 ± 13.38 Survival:71.46 ± 14.66	1 d, 4 d	D1:30.33(19.61,46.50) D4:28.58(14.83,40.62)	Shijiazhuang, China
Zhu 2023	NA	86 (51/35)	100 (54/46)	SAE:55.45 ± 6.71 NE:55.48 ± 6.89	within 24 h	9.67 ± 1.03	Changsha, China

SAE, sepsis-associated encephalopathy; NSE, neuron-specific enolase; NE, non-encephalopathy sepsis.

### 3.2 Quality assessment and publication bias

**Figure 2:**
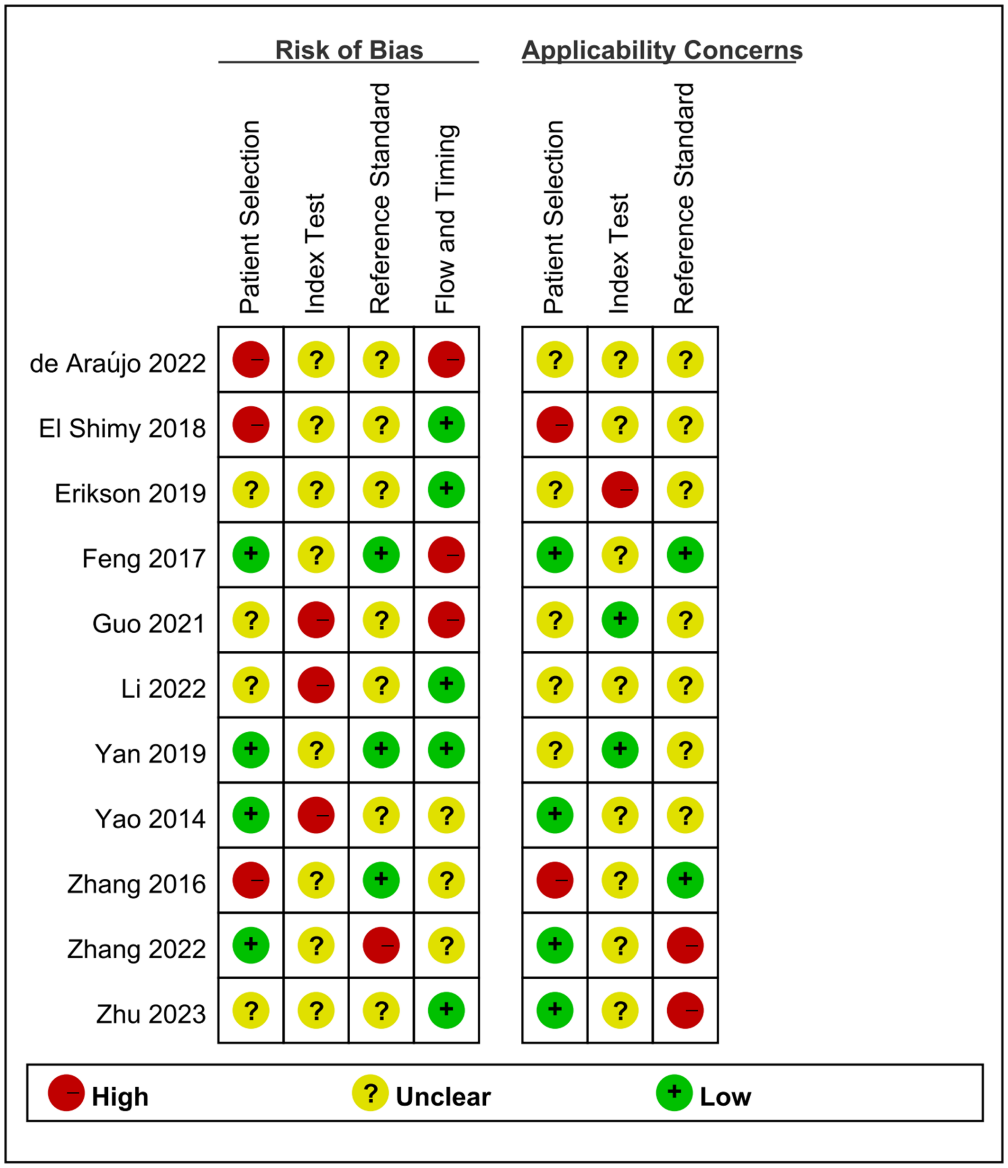
Methodological quality summary.

**Figure 3:**
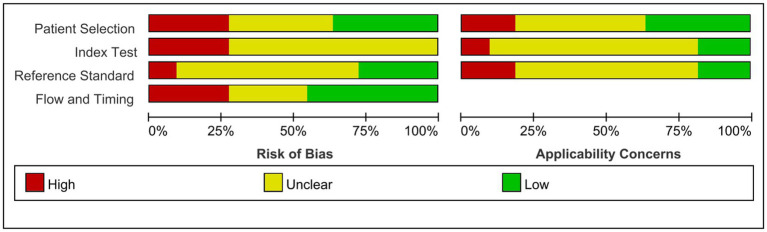
Methodological quality graph.

The quality and publication bias risk of the 11 studies were assessed by the new QUADAS-2 assessment tool, and the results are shown in Figures 2, 3. Four studies were evaluated as having a low risk of bias in patient selection, eight were evaluated as having an unclear risk in index testing, seven were evaluated as having an unclear risk in reference standards, and five were evaluated as having a low risk of bias in flow and time risks. Four studies paid little attention to patient selection, two paid little attention to index tests, and two paid little attention to reference standards. More details are shown in the Supplementary Table S1. In summary, the high-risk parameters were referred to index tests and reference standards.

### 3.3 Outcomes

#### 3.3.1 Comparison of serum NSE levels between SAE and NE

**Figure 4:**
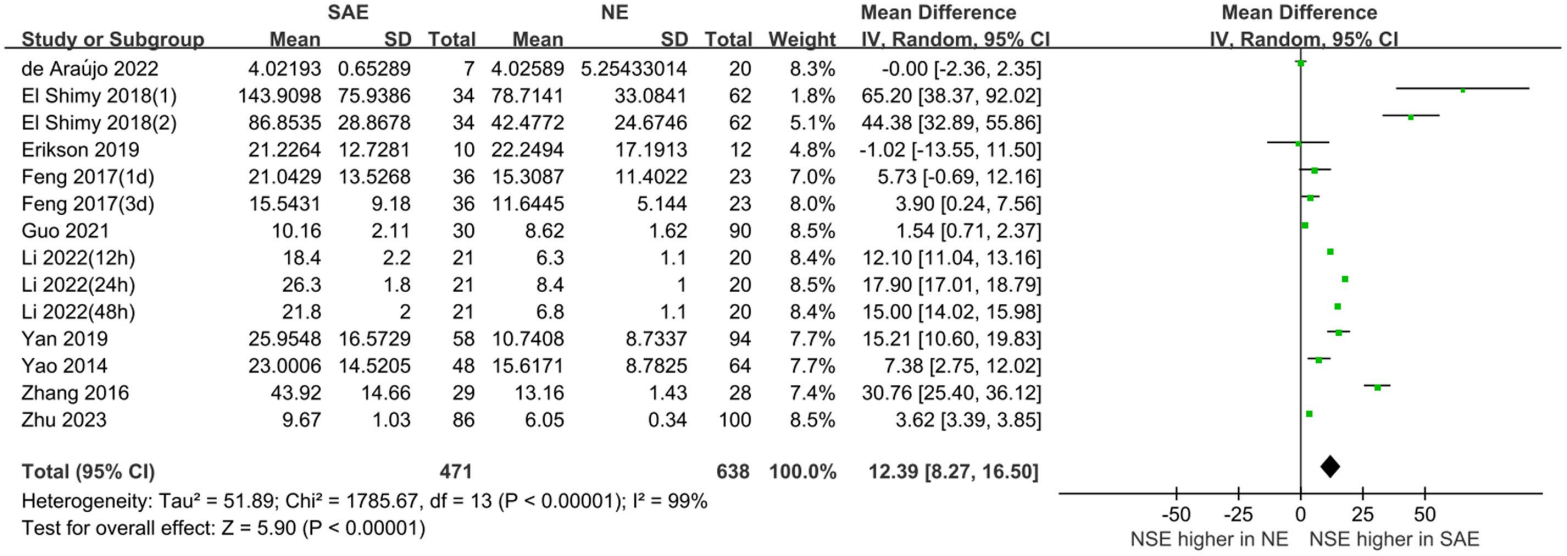
Meta-analysis forest plot: relation between serum neuron-specific enolase level and patients with sepsis-associated encephalopathy.

Serum NSE levels were compared between patients with SAE in the experimental group and those with NE in the control group, which are shown in Figure 4. The heterogeneity test indicated severe heterogeneity (*I*^2^ = 99% > 50%); thus, the random-effects model was selected. The STATA17.0 (StataCorp, College Station, TX) software analysis indicated that the pooled MD was 12.39 [95% CI 8.27–16.50, *p* < 0.00001]. Moreover, the results indicated that serum NSE levels were higher in patients with SAE than in those with NE. Furthermore, the difference in serum NSE levels between the experimental and control groups was significant (*p* < 0.05), which suggests that serum NSE may be used as a valuable biomarker of SAE to assist in clinical diagnosis.

#### 3.3.2 Comparison of serum NSE levels between patients with different outcomes

**Figure 5:**
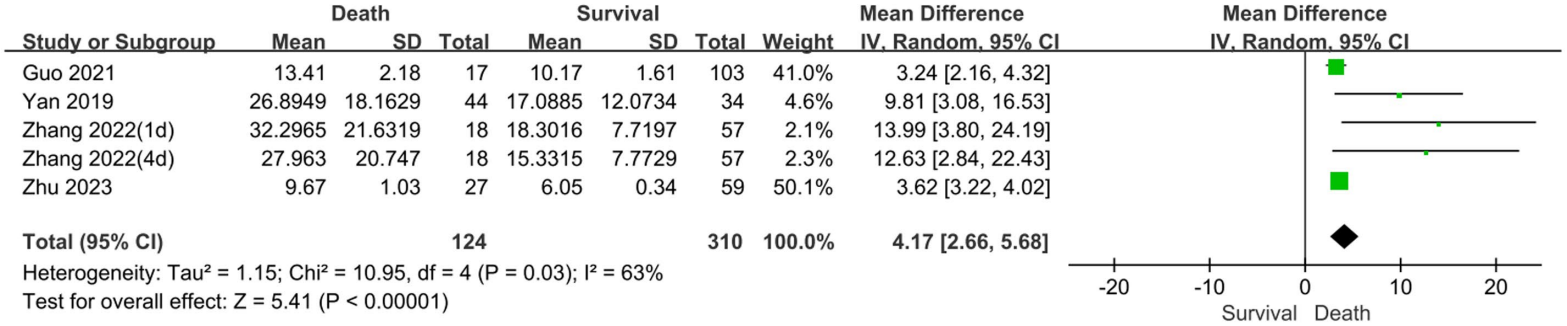
Meta-analysis forest plot: relation between serum neuron-specific enolase level and poor outcomes of patients with sepsis.

The prognostic assessment outcomes were death and survival. Four studies reported differences in serum NSE levels between patients in the death and survival groups. Figure 5 shows the results of the MD comparative analysis of serum NSE levels between the experimental and control groups. The heterogeneity test indicated severe heterogeneity (*I*^2^ = 63% > 50%); thus, a random-effects model was selected. The pooled MD was 4.17 [95% CI 2.66–5.68, *p* < 0.00001], and serum NSE levels were much higher in the death than in the survival group. Therefore, elevated serum NSE levels may predict poor prognosis in patients with sepsis. These results provide a new potential serum biomarker for determining the prognosis of sepsis, which warrants further investigation.

### 3.4 Subgroup analysis

**Figure 6:**
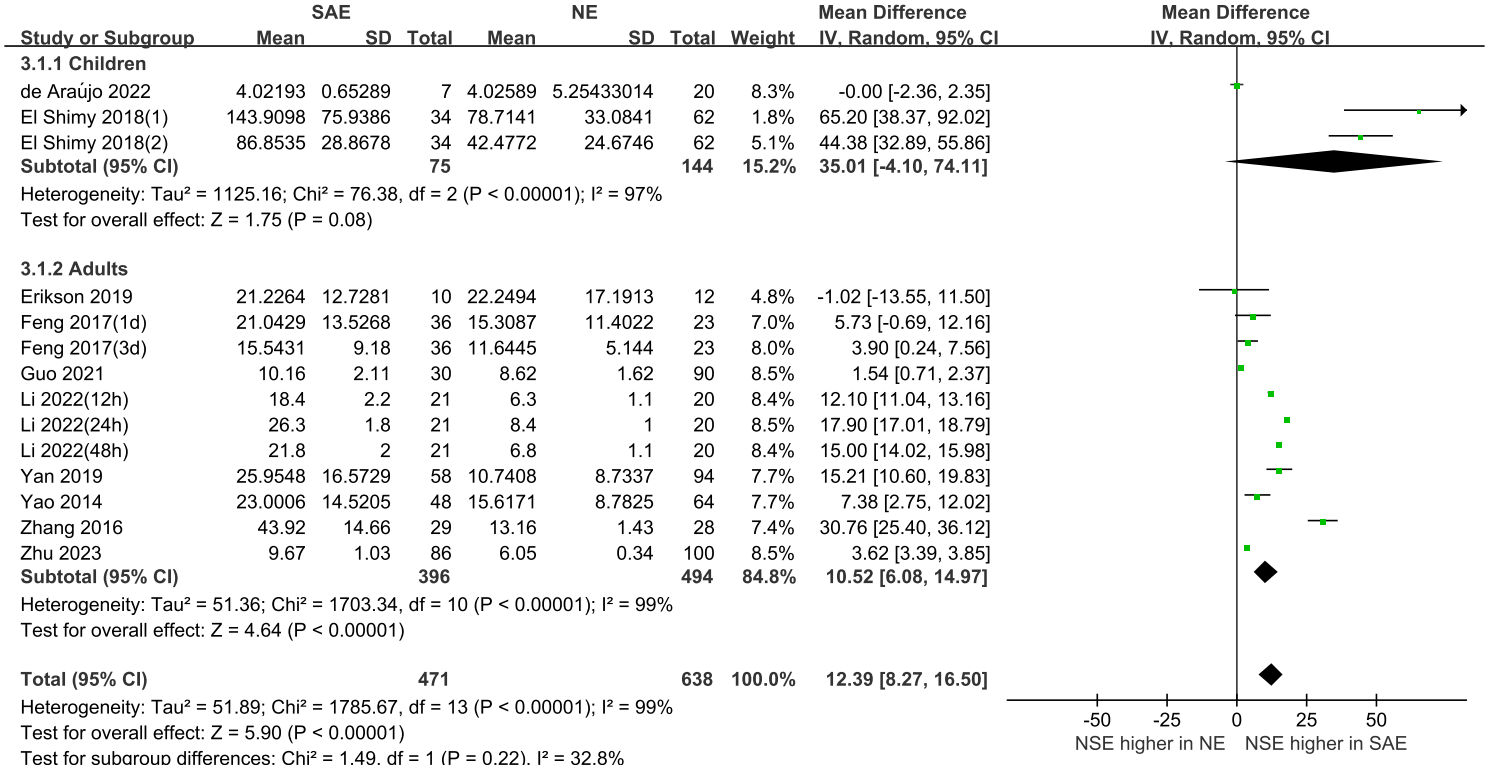
Relation between serum neuron-specific enolase levels and sepsis-associated encephalopathy at various serum sample collection times following intensive care unit admission.

**Figure 7:**
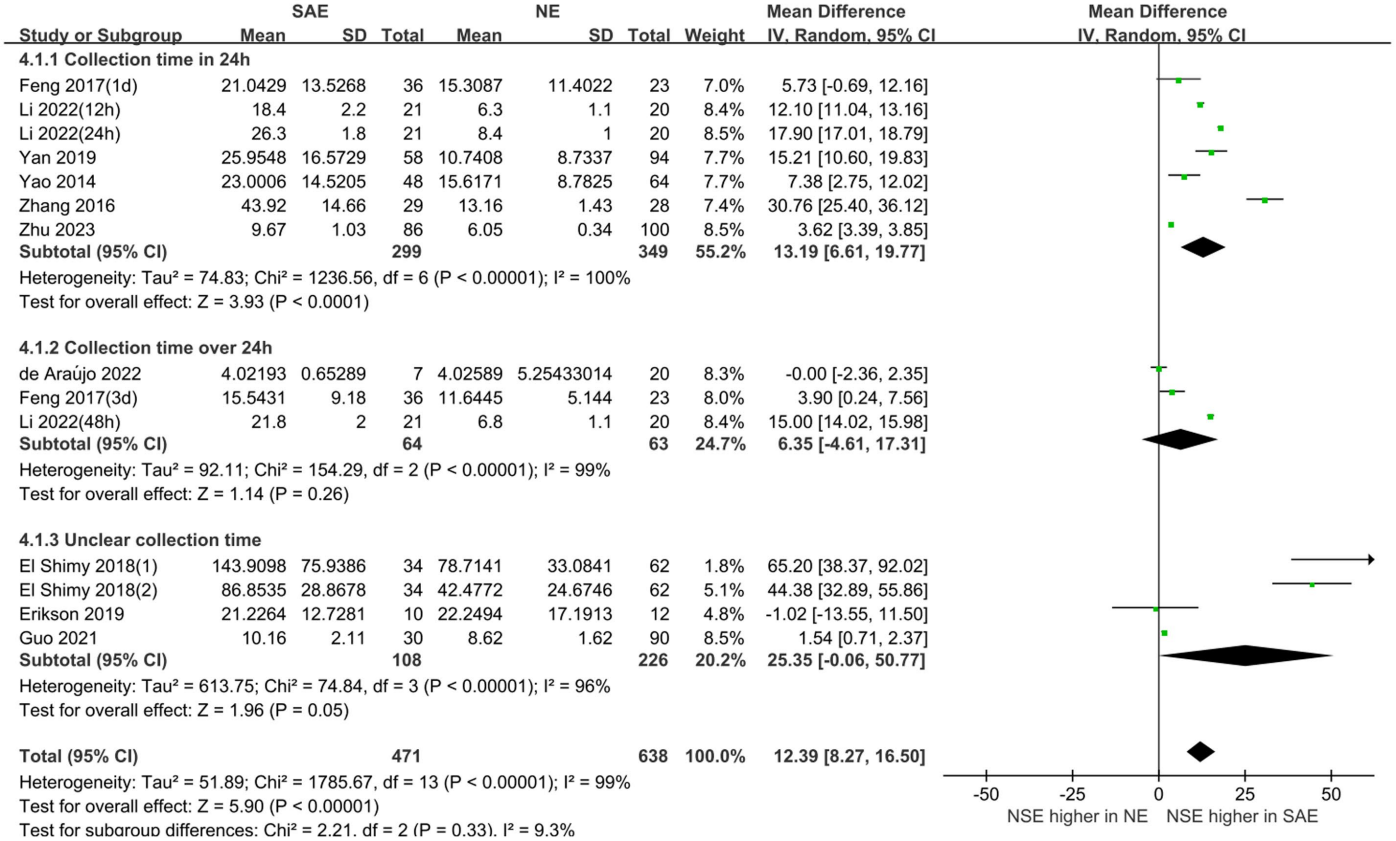
Relation between serum neuron-specific enolase level and sepsis-associated encephalopathy of varying ages.

Subgroup analyses were performed for different ages and serum collection times to explore the effects on the meta-analysis results. Figures 6, 7 show that different age cohorts (children: *I*^2^ = 97%, *p* < 0.00001; adults: *I*^2^ = 99%, *p* < 0.00001), and serum collection times (collection time ≤ 24 h: *I*^2^ = 100%, *p* < 0.00001; collection time > 24 h: *I*^2^ = 99%, *p* < 0.00001; collection time unknown: *I*^2^ = 96%, *p* < 0.00001) did not cause heterogeneity. The results in Figure 6 indicate that the difference in serum NSE levels in children was not significant (MD, 35.01 [95% CI 4.10–74.11, *Z* = 1.75, *p* = 0.08]), whereas the difference in adults was significant (MD, 10.52 [95% CI 6.08–14.97, *Z* = 4.64, *p* < 0.00001]). Figure 7 shows that the difference in serum NSE levels was only significant among the samples collected within 24 h (MD, 13.19 [95% CI 6.61–19.77, *Z* = 3.93, *p* < 0.0001]), and there was no significant difference in other groups over 24 h (MD, 6.35 [95% CI −4.61–17.31, *Z* = 1.14, *p* = 0.26]) or those with an unclear collection time (MD, 25.35 [95% CI −0.06–50.77, *Z* = 1.96, *p* = 0.05]). In addition, the comprehensive results of the subgroup analysis were significant, which was in line with the overall comprehensive results. Regardless of when serum samples were collected (MD, 12.39 [95% CI 8.27–16.50, *Z* = 5.90, *p* < 0.00001]) and at what age (MD, 12.39 [95% CI 8.27–16.50, *Z* = 5.90, *p* < 0.00001]), the SAE experimental group’s serum NSE levels were significantly higher than those in the NE group.

### 3.5 Publication bias and sensitivity analysis

**Figure 8:**
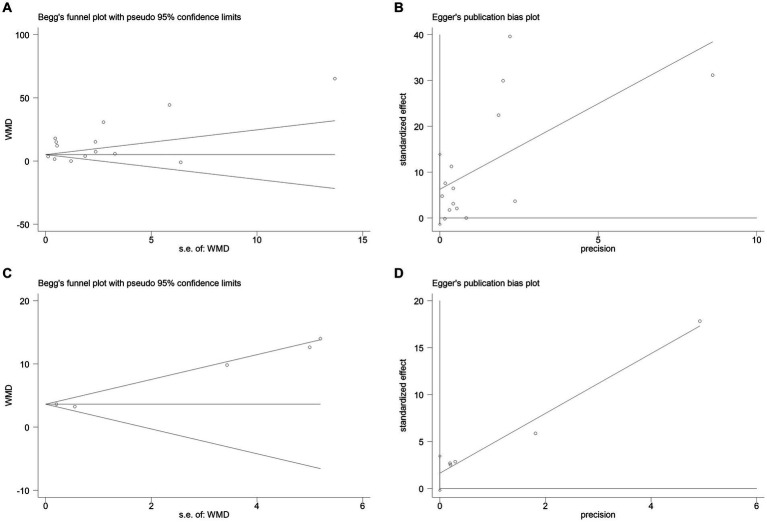
**(A)** Begg’s funnel plot analysis of the studies that investigated the relation between serum neuron-specific enolase (NSE) level and sepsis-associated encephalopathy (SAE). **(B)** Egger’s funnel plot analysis of the studies that investigated the relation between serum NSE level and SAE. **(C)** Begg’s funnel plot analysis of the studies that investigated the relation between serum NSE level and poor outcomes of patients with sepsis. **(D)** Egger’s funnel plot analysis of studies that investigated the relation between serum NSE level and poor outcomes of patients with sepsis.

**Figure 9:**
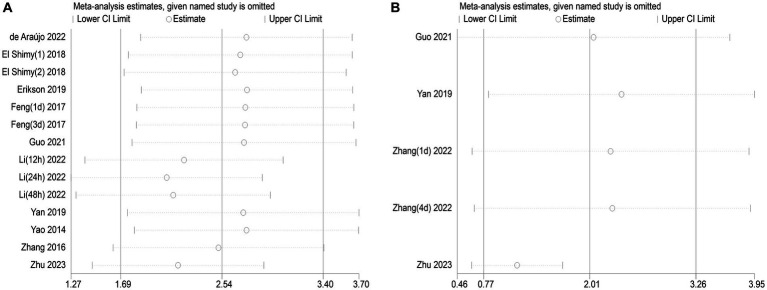
**(A)** Impact of a single study on combined mean deviation (MD) regarding the connection between serum neuron-specific enolase (NSE) level and sepsis-associated encephalopathy. **(B)** Impact of a single study on combined MD regarding the connection between serum NSE levels and the poor outcomes of patients with sepsis.

The publication bias in the included studies was evaluated using Egger’s and Begg’s regression tests. When evaluating the relationship between serum NSE and SAE, the results of Egger’s (*t* = 1.79, *p* = 0.099) and Begg’s (*Z* = 0.22, *p* = 0.827) tests indicated no significant publication bias in the included literature (Figures 8A,B). Egger’s (*t* = 2.84, *p* = 0.065) and Begg’s (*Z* = 1.22, *p* = 0.221) tests showed that there was no significant publication bias in the included studies when evaluating the relationship between serum NSE levels and the prognosis of sepsis patients (Figures 8C,D). In addition, the sensitivity analysis revealed that the study by Zhu et al. (32) had a significant impact on the results of both analyses (Figures 9A,B), and the study by Li et al. (27) had a significant effect on the results of the NSE diagnosis of SAE (Figure 9A). In addition, the results of this study were not significantly influenced by any of the other individual studies; consequently, the findings of our meta-analysis are relatively stable.

## 4 Discussion

SAE is one of the most common complications of sepsis (33); however, the lack of accurate and uniform diagnostic criteria for SAE has limited the treatment and risk management of patients in the ICU. Therefore, an accurate and reliable diagnostic method for early SAE detection is urgently needed. NSE, as a potential candidate biomarker of brain injury, has a long half-life and persistently high levels reflect brain inflammation and neuronal death to some extent (34, 35). Furthermore, measuring blood markers is easier and cheaper than other modalities (e.g., magnetic resonance imaging, EEG) (12).

**Figure 10:**
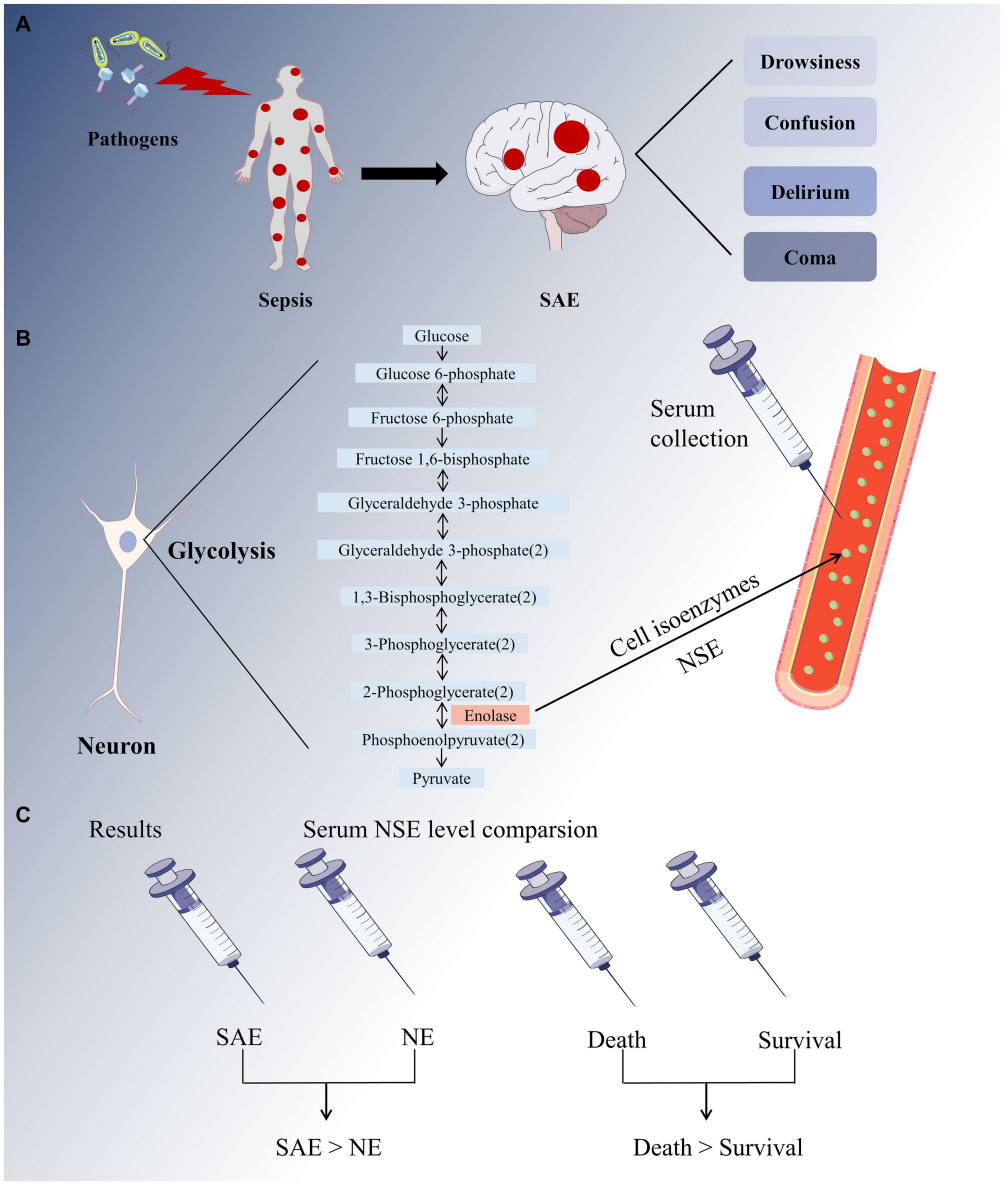
A full-text overview picture. **(A)** The invasion of pathogens into the human body causes systemic inflammatory response, resulting in sepsis. The major sepsis-associated encephalopathy (SAE) symptoms range from drowsiness to confusion, delirium, and coma. **(B)** Neuron-specific enolase (NSE) is a cell isoenzyme of enolase in glycolysis that can be detected in serum. **(C)** Our meta-analysis revealed that the SAE group had higher serum NSE levels than that in the NE group, and the sepsis mortality group had higher serum NSE levels than the survival group.

To the best of our knowledge, this is the first comprehensive meta-analysis to evaluate the diagnostic and prognostic value of serum NSE as a biomarker for SAE (Figure 10). First, we used strict screening criteria to select studies for inclusion in our analysis. To obtain more insight into the data from these studies, we placed no restrictions on the type of observational study and included prospective and retrospective studies. Second, the QUADAS-2 tool was used to conduct a comprehensive quality and bias assessment. Notably, according to our meta-analysis, patients with SAE had higher serum NSE levels than patients with NE, as did those in the poor prognosis and sepsis survival groups. Meanwhile, the findings of two of the studies that were not included in the analysis due to data retrieval difficulties are also supportive of the analyses derived herein. The result of one study demonstrated that for each twofold increase in plasma NSE concentration, there was a 5.2% (95% CI 3.2–7.2, *p* < 0.001) increase in the risk of delirium and a 7.3% (95% CI 2.5–12.0, *p* = 0.003) (36). Another study concluded that patients who died within the first 4 days (early deaths) had higher NSE values compared to patients who died later (late deaths) and survivors (37). Therefore, serum NSE may be a promising SAE biomarker.

Owing to the large heterogeneity of the included studies, subgroup and sensitivity analyses were conducted. Differences in NSE levels in children were not significant in the subgroup analyses, due to an insufficient sample size or number of studies. However, the differences in NSE levels in adults were found to be significant in the subgroup analyses. Simultaneously, subgroup heterogeneity of the adults and children was high, thus excluding age as a source of heterogeneity. The findings of another subgroup analysis indicated that serum NSE levels measured within 24 h after ICU admission significantly differed in SAE and NE. Therefore, it is recommended for relevant clinical studies to collect serum samples at multiple earlier time periods to better detect NSE. Furthermore, the heterogeneity of each subgroup time was also high, thus excluding serum sampling time. The sensitivity analysis results indicated that the studies by Li et al. (27) and Zhu et al. (32) may have led to heterogeneity. The SAE patients included in the study by Li et al. (27) were burn patients with complex medical conditions and were quite heterogeneous compared to other patients included in the studies analyzed for NSE diagnosis. The sample size in that study was also small, with a total sample size of only 41 cases. As such, these two factors are likely to be the possible reasons why this study has a significant impact on the diagnostic results of NSE. The unclear diagnostic criteria of the SAE patients included in the study by Zhu et al. (32) may have contributed to the significant impact of this study on the analyzed results. Overall, the results of our meta-analysis were statistically stable.

Notably, although the results of our analysis showed no significant difference between the SAE and NE groups after 24 h, it does not exclude the diagnostic efficacy of NSE for SAE over 24 h. This may be related to the small sample size of studies we included in the over-24 h analysis and the large heterogeneity (up to 99%) among the studies. In addition to this, we found from studies of other diseases that NSE after 72 h was associated with mortality and poor functional prognosis in patients under venoarterial extracorporeal membrane oxygenation (38) as well as in cardiac arrest (39). Therefore, more multicenter large-sample studies are still needed to explore the prognostic role of NSE for prolonged periods of time (>24 h).

Our meta-analysis had the following limitations. First, only four studies on the relationship between NSE and sepsis prognosis were included in the meta-analysis. Second, the heterogeneity of the studies was high, and the subgroup analyses failed to reveal its source; we speculate that this heterogeneity may stem from different sepsis diagnostic criteria, regional factors, and different serum NSE detection methods. Finally, our analysis did not discriminate by type of study – we included prospective and retrospective studies. This limitation was unavoidable, owing to the restricted number of studies that fit the inclusion criteria.

Taken together, our results suggest that serum NSE levels, as a clinical indicator to evaluate the diagnosis of SAE and survival outcomes in patients with sepsis, merit further investigation. In addition, it is worth noting that NSE is also present in red blood cells and platelets. Therefore, it is necessary to exclude hemolytic samples when measuring NSE in plasma or serum to avoid NSE interference results from other sources (40). Further studies are required to find the optimal combination of biomarkers for diagnostic and prognostic purposes. The optimal combination of diagnostic sensitivity and specificity of serological markers, along with the appropriate diagnostic methods, such as EEG, computed tomography, and magnetic resonance imaging, also deserves to be explored. In addition, investigating whether NSE-related molecular loci provide targets for SAE therapy may be worthwhile.

In conclusion, higher serum NSE levels moderately correlated with SAE and poor prognosis. Serum NSE level may be a potential biomarker for the diagnosis and prognosis of SAE.

## Data availability statement

The original contributions presented in the study are included in the article/Supplementary material, further inquiries can be directed to the corresponding authors.

## Author contributions

MP: Conceptualization, Writing – original draft. YY: Data curation, Methodology, Writing – original draft. CZ: Formal analysis, Software, Writing – original draft. QH: Investigation, Writing – original draft. YF: Visualization, Writing – original draft. LX: Resources, Supervision, Writing – original draft. SL: Funding acquisition, Writing – review & editing. HH: Funding acquisition, Project administration, Writing – review & editing.
