# Supplementary material for: Role of serum neuron-specific enolase levels in the early diagnosis and prognosis of sepsis-associated encephalopathy: a systematic review and meta-analysis
Source: Front Neurol. 2024 Feb 29;15:1353063. doi: 10.3389/fneur.2024.1353063 (PMC11057363; doi:10.3389/fneur.2024.1353063)
Supplement: Supplementary file 1 [file Table_1.docx]

**QUADAS 2**

| **Study and year** | **Item** | **Assessment** |
| --- | --- | --- |
| **de Araújo 2022** | **Domain 1: Patient selection** |  |
|  | **A. Risk of Bias** |  |
|  | Was a consecutive or random sample of patients enrolled? | No |
|  | Was a case-control design avoided? | Yes |
|  | Did the study avoid inappropriate exclusions? | Unclear |
|  | Could the selection of patients have introduced bias? | High risk |
|  | **B. Concerns regarding applicability** |  |
|  | Are there concerns that the included patients and setting do not match the review question? | Unclear concern |
|  | **Domain 2: Index test** |  |
|  | **A. Risk of Bias** |  |
|  | Were the index test results interpreted without knowledge of the results of the reference standard? | Yes |
|  | If a threshold was used, was it pre-specified? | Unclear |
|  | Could the conduct or interpretation of the index test have introduced bias? | Unclear risk |
|  | **B. Concerns regarding applicability** |  |
|  | Are there concerns that the index test, its conduct, or interpretation differ from the review question? | Unclear concern |
|  | **Domain 3: Reference standard** |  |
|  | **A. Risk of Bias** |  |
|  | Is the reference standards likely to correctly classify the target condition? | Unclear |
|  | Were the reference standard results interpreted without knowledge of the results of the index tests? | Unclear |
|  | Could the reference standard, its conduct, or its interpretation have introduced bias? | Unclear risk |
|  | **B. Concerns regarding applicability** |  |
|  | Are there concerns that the target condition as defined by the reference standard does not match the question? | Unclear concern |
|  | **Domain 4: Flow and timing** |  |
|  | **A. Risk of Bias** |  |
|  | Was there an appropriate interval between index test and reference standard? | Unclear |
|  | Did all patients receive the same reference standard? | Yes |
|  | Were all patients included in the analysis? | No |
|  | Could the patient flow have introduced bias? | High risk |
| **El Shimy 2018** | **Domain 1: Patient selection** |  |
|  | **A. Risk of Bias** |  |
|  | Was a consecutive or random sample of patients enrolled? | Yes |
|  | Was a case-control design avoided? | Yes |
|  | Did the study avoid inappropriate exclusions? | No |
|  | Could the selection of patients have introduced bias? | High risk |
|  | **B. Concerns regarding applicability** |  |
|  | Are there concerns that the included patients and setting do not match the review question? | High concern |
|  | **Domain 2: Index test** |  |
|  | **A. Risk of Bias** |  |
|  | Were the index test results interpreted without knowledge of the results of the reference standard? | Yes |
|  | If a threshold was used, was it pre-specified? | Unclear |
|  | Could the conduct or interpretation of the index test have introduced bias? | Unclear risk |
|  | **B. Concerns regarding applicability** |  |
|  | Are there concerns that the index test, its conduct, or interpretation differ from the review question? | Unclear concern |
|  | **Domain 3: Reference standard** |  |
|  | **A. Risk of Bias** |  |
|  | Is the reference standards likely to correctly classify the target condition? | Unclear |
|  | Were the reference standard results interpreted without knowledge of the results of the index tests? | Yes |
|  | Could the reference standard, its conduct, or its interpretation have introduced bias? | Unclear risk |
|  | **B. Concerns regarding applicability** |  |
|  | Are there concerns that the target condition as defined by the reference standard does not match the question? | Unclear concern |
|  | **Domain 4: Flow and timing** |  |
|  | **A. Risk of Bias** |  |
|  | Was there an appropriate interval between index test and reference standard? | Yes |
|  | Did all patients receive the same reference standard? | Yes |
|  | Were all patients included in the analysis? | Yes |
|  | Could the patient flow have introduced bias? | Low risk |
| **Erikson 2019** | **Domain 1: Patient selection** |  |
|  | **A. Risk of Bias** |  |
|  | Was a consecutive or random sample of patients enrolled? | Unclear |
|  | Was a case-control design avoided? | Unclear |
|  | Did the study avoid inappropriate exclusions? | Yes |
|  | Could the selection of patients have introduced bias? | Unclear risk |
|  | **B. Concerns regarding applicability** |  |
|  | Are there concerns that the included patients and setting do not match the review question? | Unclear concern |
|  | **Domain 2: Index test** |  |
|  | **A. Risk of Bias** |  |
|  | Were the index test results interpreted without knowledge of the results of the reference standard? | Yes |
|  | If a threshold was used, was it pre-specified? | Unclear |
|  | Could the conduct or interpretation of the index test have introduced bias? | Unclear risk |
|  | **B. Concerns regarding applicability** |  |
|  | Are there concerns that the index test, its conduct, or interpretation differ from the review question? | High concern |
|  | **Domain 3: Reference standard** |  |
|  | **A. Risk of Bias** |  |
|  | Is the reference standards likely to correctly classify the target condition? | Yes |
|  | Were the reference standard results interpreted without knowledge of the results of the index tests? | Unclear |
|  | Could the reference standard, its conduct, or its interpretation have introduced bias? | Unclear risk |
|  | **B. Concerns regarding applicability** |  |
|  | Are there concerns that the target condition as defined by the reference standard does not match the question? | Unclear concern |
|  | **Domain 4: Flow and timing** |  |
|  | **A. Risk of Bias** |  |
|  | Was there an appropriate interval between index test and reference standard? | Yes |
|  | Did all patients receive the same reference standard? | Yes |
|  | Were all patients included in the analysis? | Yes |
|  | Could the patient flow have introduced bias? | Low risk |
| **Feng 2017** | **Domain 1: Patient selection** |  |
|  | **A. Risk of Bias** |  |
|  | Was a consecutive or random sample of patients enrolled? | Yes |
|  | Was a case-control design avoided? | Yes |
|  | Did the study avoid inappropriate exclusions? | Yes |
|  | Could the selection of patients have introduced bias? | Low risk |
|  | **B. Concerns regarding applicability** |  |
|  | Are there concerns that the included patients and setting do not match the review question? | Low concern |
|  | **Domain 2: Index test** |  |
|  | **A. Risk of Bias** |  |
|  | Were the index test results interpreted without knowledge of the results of the reference standard? | Unclear |
|  | If a threshold was used, was it pre-specified? | Yes |
|  | Could the conduct or interpretation of the index test have introduced bias? | Unclear risk |
|  | **B. Concerns regarding applicability** |  |
|  | Are there concerns that the index test, its conduct, or interpretation differ from the review question? | Unclear concern |
|  | **Domain 3: Reference standard** |  |
|  | **A. Risk of Bias** |  |
|  | Is the reference standards likely to correctly classify the target condition? | Yes |
|  | Were the reference standard results interpreted without knowledge of the results of the index tests? | Yes |
|  | Could the reference standard, its conduct, or its interpretation have introduced bias? | Low risk |
|  | **B. Concerns regarding applicability** |  |
|  | Are there concerns that the target condition as defined by the reference standard does not match the question? | Low concern |
|  | **Domain 4: Flow and timing** |  |
|  | **A. Risk of Bias** |  |
|  | Was there an appropriate interval between index test and reference standard? | Unclear |
|  | Did all patients receive the same reference standard? | Yes |
|  | Were all patients included in the analysis? | No |
|  | Could the patient flow have introduced bias? | High risk |
| **Guo 2021** | **Domain 1: Patient selection** |  |
|  | **A. Risk of Bias** |  |
|  | Was a consecutive or random sample of patients enrolled? | Yes |
|  | Was a case-control design avoided? | Unclear |
|  | Did the study avoid inappropriate exclusions? | Yes |
|  | Could the selection of patients have introduced bias? | Unclear risk |
|  | **B. Concerns regarding applicability** |  |
|  | Are there concerns that the included patients and setting do not match the review question? | Unclear concern |
|  | **Domain 2: Index test** |  |
|  | **A. Risk of Bias** |  |
|  | Were the index test results interpreted without knowledge of the results of the reference standard? | Unclear |
|  | If a threshold was used, was it pre-specified? | No |
|  | Could the conduct or interpretation of the index test have introduced bias? | High risk |
|  | **B. Concerns regarding applicability** |  |
|  | Are there concerns that the index test, its conduct, or interpretation differ from the review question? | Low concern |
|  | **Domain 3: Reference standard** |  |
|  | **A. Risk of Bias** |  |
|  | Is the reference standards likely to correctly classify the target condition? | Unclear |
|  | Were the reference standard results interpreted without knowledge of the results of the index tests? | Yes |
|  | Could the reference standard, its conduct, or its interpretation have introduced bias? | Unclear risk |
|  | **B. Concerns regarding applicability** |  |
|  | Are there concerns that the target condition as defined by the reference standard does not match the question? | Unclear concern |
|  | **Domain 4: Flow and timing** |  |
|  | **A. Risk of Bias** |  |
|  | Was there an appropriate interval between index test and reference standard? | Unclear |
|  | Did all patients receive the same reference standard? | No |
|  | Were all patients included in the analysis? | Yes |
|  | Could the patient flow have introduced bias? | High risk |
| **Li 2022** | **Domain 1: Patient selection** |  |
|  | **A. Risk of Bias** |  |
|  | Was a consecutive or random sample of patients enrolled? | Yes |
|  | Was a case-control design avoided? | Unclear |
|  | Did the study avoid inappropriate exclusions? | Yes |
|  | Could the selection of patients have introduced bias? | Unclear risk |
|  | **B. Concerns regarding applicability** |  |
|  | Are there concerns that the included patients and setting do not match the review question? | Unclear concern |
|  | **Domain 2: Index test** |  |
|  | **A. Risk of Bias** |  |
|  | Were the index test results interpreted without knowledge of the results of the reference standard? | No |
|  | If a threshold was used, was it pre-specified? | Unclear |
|  | Could the conduct or interpretation of the index test have introduced bias? | High risk |
|  | **B. Concerns regarding applicability** |  |
|  | Are there concerns that the index test, its conduct, or interpretation differ from the review question? | Unclear concern |
|  | **Domain 3: Reference standard** |  |
|  | **A. Risk of Bias** |  |
|  | Is the reference standards likely to correctly classify the target condition? | Unclear |
|  | Were the reference standard results interpreted without knowledge of the results of the index tests? | Yes |
|  | Could the reference standard, its conduct, or its interpretation have introduced bias? | Unclear risk |
|  | **B. Concerns regarding applicability** |  |
|  | Are there concerns that the target condition as defined by the reference standard does not match the question? | Unclear concern |
|  | **Domain 4: Flow and timing** |  |
|  | **A. Risk of Bias** |  |
|  | Was there an appropriate interval between index test and reference standard? | Yes |
|  | Did all patients receive the same reference standard? | Yes |
|  | Were all patients included in the analysis? | Yes |
|  | Could the patient flow have introduced bias? | Low risk |
| **Yan 2019** | **Domain 1: Patient selection** |  |
|  | **A. Risk of Bias** |  |
|  | Was a consecutive or random sample of patients enrolled? | Yes |
|  | Was a case-control design avoided? | Yes |
|  | Did the study avoid inappropriate exclusions? | Yes |
|  | Could the selection of patients have introduced bias? | Low risk |
|  | **B. Concerns regarding applicability** |  |
|  | Are there concerns that the included patients and setting do not match the review question? | Unclear concern |
|  | **Domain 2: Index test** |  |
|  | **A. Risk of Bias** |  |
|  | Were the index test results interpreted without knowledge of the results of the reference standard? | Yes |
|  | If a threshold was used, was it pre-specified? | Unclear |
|  | Could the conduct or interpretation of the index test have introduced bias? | Unclear risk |
|  | **B. Concerns regarding applicability** |  |
|  | Are there concerns that the index test, its conduct, or interpretation differ from the review question? | Low concern |
|  | **Domain 3: Reference standard** |  |
|  | **A. Risk of Bias** |  |
|  | Is the reference standards likely to correctly classify the target condition? | Yes |
|  | Were the reference standard results interpreted without knowledge of the results of the index tests? | Yes |
|  | Could the reference standard, its conduct, or its interpretation have introduced bias? | Low risk |
|  | **B. Concerns regarding applicability** |  |
|  | Are there concerns that the target condition as defined by the reference standard does not match the question? | Unclear concern |
|  | **Domain 4: Flow and timing** |  |
|  | **A. Risk of Bias** |  |
|  | Was there an appropriate interval between index test and reference standard? | Yes |
|  | Did all patients receive the same reference standard? | Yes |
|  | Were all patients included in the analysis? | Yes |
|  | Could the patient flow have introduced bias? | Low risk |
| **Yao 2014** | **Domain 1: Patient selection** |  |
|  | **A. Risk of Bias** |  |
|  | Was a consecutive or random sample of patients enrolled? | Yes |
|  | Was a case-control design avoided? | Yes |
|  | Did the study avoid inappropriate exclusions? | Yes |
|  | Could the selection of patients have introduced bias? | Low risk |
|  | **B. Concerns regarding applicability** |  |
|  | Are there concerns that the included patients and setting do not match the review question? | Low concern |
|  | **Domain 2: Index test** |  |
|  | **A. Risk of Bias** |  |
|  | Were the index test results interpreted without knowledge of the results of the reference standard? | No |
|  | If a threshold was used, was it pre-specified? | Unclear |
|  | Could the conduct or interpretation of the index test have introduced bias? | High risk |
|  | **B. Concerns regarding applicability** |  |
|  | Are there concerns that the index test, its conduct, or interpretation differ from the review question? | Unclear concern |
|  | **Domain 3: Reference standard** |  |
|  | **A. Risk of Bias** |  |
|  | Is the reference standards likely to correctly classify the target condition? | Unclear |
|  | Were the reference standard results interpreted without knowledge of the results of the index tests? | Yes |
|  | Could the reference standard, its conduct, or its interpretation have introduced bias? | Unclear risk |
|  | **B. Concerns regarding applicability** |  |
|  | Are there concerns that the target condition as defined by the reference standard does not match the question? | Unclear concern |
|  | **Domain 4: Flow and timing** |  |
|  | **A. Risk of Bias** |  |
|  | Was there an appropriate interval between index test and reference standard? | Yes |
|  | Did all patients receive the same reference standard? | Unclear |
|  | Were all patients included in the analysis? | Yes |
|  | Could the patient flow have introduced bias? | Unclear risk |
| **Zhang 2016** | **Domain 1: Patient selection** |  |
|  | **A. Risk of Bias** |  |
|  | Was a consecutive or random sample of patients enrolled? | Yes |
|  | Was a case-control design avoided? | No |
|  | Did the study avoid inappropriate exclusions? | Yes |
|  | Could the selection of patients have introduced bias? | High risk |
|  | **B. Concerns regarding applicability** |  |
|  | Are there concerns that the included patients and setting do not match the review question? | High concern |
|  | **Domain 2: Index test** |  |
|  | **A. Risk of Bias** |  |
|  | Were the index test results interpreted without knowledge of the results of the reference standard? | Yes |
|  | If a threshold was used, was it pre-specified? | Unclear |
|  | Could the conduct or interpretation of the index test have introduced bias? | Unclear risk |
|  | **B. Concerns regarding applicability** |  |
|  | Are there concerns that the index test, its conduct, or interpretation differ from the review question? | Unclear concern |
|  | **Domain 3: Reference standard** |  |
|  | **A. Risk of Bias** |  |
|  | Is the reference standards likely to correctly classify the target condition? | Yes |
|  | Were the reference standard results interpreted without knowledge of the results of the index tests? | Yes |
|  | Could the reference standard, its conduct, or its interpretation have introduced bias? | Low risk |
|  | **B. Concerns regarding applicability** |  |
|  | Are there concerns that the target condition as defined by the reference standard does not match the question? | Low concern |
|  | **Domain 4: Flow and timing** |  |
|  | **A. Risk of Bias** |  |
|  | Was there an appropriate interval between index test and reference standard? | Unclear |
|  | Did all patients receive the same reference standard? | Yes |
|  | Were all patients included in the analysis? | Yes |
|  | Could the patient flow have introduced bias? | Unclear risk |
| **Zhang 2022** | **Domain 1: Patient selection** |  |
|  | **A. Risk of Bias** |  |
|  | Was a consecutive or random sample of patients enrolled? | Yes |
|  | Was a case-control design avoided? | Yes |
|  | Did the study avoid inappropriate exclusions? | Yes |
|  | Could the selection of patients have introduced bias? | Low risk |
|  | **B. Concerns regarding applicability** |  |
|  | Are there concerns that the included patients and setting do not match the review question? | Low concern |
|  | **Domain 2: Index test** |  |
|  | **A. Risk of Bias** |  |
|  | Were the index test results interpreted without knowledge of the results of the reference standard? | Yes |
|  | If a threshold was used, was it pre-specified? | Unclear |
|  | Could the conduct or interpretation of the index test have introduced bias? | Unclear risk |
|  | **B. Concerns regarding applicability** |  |
|  | Are there concerns that the index test, its conduct, or interpretation differ from the review question? | Unclear concern |
|  | **Domain 3: Reference standard** |  |
|  | **A. Risk of Bias** |  |
|  | Is the reference standards likely to correctly classify the target condition? | Yes |
|  | Were the reference standard results interpreted without knowledge of the results of the index tests? | No |
|  | Could the reference standard, its conduct, or its interpretation have introduced bias? | High risk |
|  | **B. Concerns regarding applicability** |  |
|  | Are there concerns that the target condition as defined by the reference standard does not match the question? | High concern |
|  | **Domain 4: Flow and timing** |  |
|  | **A. Risk of Bias** |  |
|  | Was there an appropriate interval between index test and reference standard? | Unclear |
|  | Did all patients receive the same reference standard? | Yes |
|  | Were all patients included in the analysis? | Yes |
|  | Could the patient flow have introduced bias? | Unclear risk |
| **Zhu 2023** | **Domain 1: Patient selection** |  |
|  | **A. Risk of Bias** |  |
|  | Was a consecutive or random sample of patients enrolled? | Yes |
|  | Was a case-control design avoided? | Yes |
|  | Did the study avoid inappropriate exclusions? | Unclear |
|  | Could the selection of patients have introduced bias? | Unclear risk |
|  | **B. Concerns regarding applicability** |  |
|  | Are there concerns that the included patients and setting do not match the review question? | Low concern |
|  | **Domain 2: Index test** |  |
|  | **A. Risk of Bias** |  |
|  | Were the index test results interpreted without knowledge of the results of the reference standard? | Yes |
|  | If a threshold was used, was it pre-specified? | Unclear |
|  | Could the conduct or interpretation of the index test have introduced bias? | Unclear risk |
|  | **B. Concerns regarding applicability** |  |
|  | Are there concerns that the index test, its conduct, or interpretation differ from the review question? | Unclear concern |
|  | **Domain 3: Reference standard** |  |
|  | **A. Risk of Bias** |  |
|  | Is the reference standards likely to correctly classify the target condition? | Unclear |
|  | Were the reference standard results interpreted without knowledge of the results of the index tests? | Unclear |
|  | Could the reference standard, its conduct, or its interpretation have introduced bias? | Unclear risk |
|  | **B. Concerns regarding applicability** |  |
|  | Are there concerns that the target condition as defined by the reference standard does not match the question? | High concern |
|  | **Domain 4: Flow and timing** |  |
|  | **A. Risk of Bias** |  |
|  | Was there an appropriate interval between index test and reference standard? | Yes |
|  | Did all patients receive the same reference standard? | Yes |
|  | Were all patients included in the analysis? | Yes |
|  | Could the patient flow have introduced bias? | Low risk |
